# Randomized, double-blind, placebo-controlled clinical trial measuring the effect of a dietetic food on dermatologic scoring and pruritus in dogs with atopic dermatitis

**DOI:** 10.1186/s12917-021-03063-w

**Published:** 2021-11-19

**Authors:** Miguel Sánchez de Santiago, José Luis González Arribas, Yolanda Moral Llamas, Iveta Becvarova, Hein Meyer

**Affiliations:** 1 Clinical Veterinary Hospital, Complutense University, Puerta de Hierro s/n, 28040 Madrid, Spain; 2 Hill’s Pet Nutrition Inc., 400 Southwest Eighth Avenue, Topeka, KS 66603 USA

**Keywords:** Canine, Atopy, Nutrition, Allergy, Dermatology, Veterinary, Diet

## Abstract

**Background:**

Canine atopic dermatitis (AD) is a common condition that often requires multimodal therapy. Including a diet in the multimodal management of AD may reduce medication doses, saving pet owners money and reducing side effects. The objective of this randomized, double-blind, placebo-controlled clinical trial was to determine if a diet fortified in antioxidants, polyphenols, and omega-3 fatty acids can reduce the clinical signs of AD. Forty client-owned dogs with AD were enrolled in the study and assigned to either an enriched diet (diet B) or control diet (diet A) for 60-days. CADESI-4 index scores and owner-reported pruritus scores were measured periodically.

**Results:**

Total CADESI-4 index scores for dogs eating diet B were lower on day 60 compared to baseline (*P* = 0.003). There was no statistical difference in scores for dogs eating diet A over a 60-day period. Diet B dogs had 25 and 49% reductions in CADESI-4 index scores on days 30 and 60, respectively (*P* = 0.0007) while diet A had no change over the study period. When comparing the percent change in owner-reported pruritus scores, diet B also performed better than diet A. By day 60, owners feeding diet B to their dogs reported a significant reduction (*P* < 0.0001) of 46.4% in itching, while those on diet A reported a 26.8% reduction, which was not statistically significant (*P* = 0.08).

**Conclusions:**

These study results demonstrate feeding a diet enriched with ingredients to improve skin health and reduce inflammation improves the clinical signs of AD in dogs.

## Background

Atopic dermatitis (AD) is a common cause of pruritus in dogs. The disease is multifactorial and defined as a “genetically predisposed inflammatory and pruritic allergic skin disease often associated with a production of immunoglobulin (Ig) E against environmental allergens” [1]. While the pathogenesis of AD is still unclear, cutaneous inflammation from an aberrant immune response and defects in the epidermal barrier play key roles [2–4].

Atopic dermatitis often requires multimodal therapy incorporating allergy avoidance, immune-modulation, and enhancement of the epidermal barrier [5]. Several studies suggest oral supplementation with polyunsaturated fatty acids (PUFAs) reduces pruritus and can lower the dosages of cyclosporine and glucocorticoids required to control clinical signs [6–10]. PUFAs are thought to combat AD by enhancing epidermal barrier function, reducing inflammatory cell activation, and altering eicosanoid production [5, 11]. In a study of healthy dogs, a diet supplemented with the omega-3 fatty acids docosahexaenoic acid (DHA) and eicosapentaenoic acid (EPA) reduced serum prostaglandin-E2 concentrations and interleukin-1 and interleukin-6 activity [12].

Dogs with AD have reduced continuity and thickness of the intracellular lipid lamella of the stratum corneum in non-lesional skin [13, 14]. In addition, dogs with AD have lower ceramide levels within the SC compared to normal controls [15, 16]. Evidence suggests oral supplementation with PUFAs can help mitigate some of these structural changes [14].

Polyphenols are naturally occurring compounds comprised of an aromatic ring with multiple phenol groups. Fruits and vegetables tend to have high polyphenol concentrations, and examples include curcumin, quercetin, resveratrol, and epigallocatechin gallate [17, 18]. Polyphenols have been studied extensively for their anti-inflammatory and anti-neoplastic properties [19–21]. There is also evidence suggesting polyphenols may aid in the treatment of allergic diseases [17, 18]. The mechanisms by which polyphenols affect allergic disease varies by class, but examples include formation of insoluble complexes with antigenic proteins to make them less allergenic [22], interference of dendritic cell function [23, 24], and reduction of B cell antibody and T cell cytokine production upon antigen re-exposure [18, 25–29]. Quercetin may also inhibit the release of mast cell inflammatory mediators and inhibit histamine synthesis [30–33].

Atopic dermatitis in both humans and dogs is linked to higher oxidative stress, likely due to a combination of lower plasma antioxidant concentrations and increased lipid peroxidation of cell membranes [34, 35]. The antioxidant vitamin E is crucial for preventing lipid peroxidation. Randomized, placebo-controlled clinical trials in humans and dogs noted improvement in clinical signs of AD with vitamin E supplementation [36, 37]. Vitamin C is another potent antioxidant and serum concentrations inversely correlate with clinical severity of AD in humans [38]. Intradermal concentrations of vitamin C are also lower in people with AD [39].

Feeding a diet high in PUFA, polyphenols, and antioxidants would provide an easy adjunct to current AD treatments. A research abstract evaluating a diet enriched with these nutrients found lower levels IL-12p40, MCP-1, and IL-2 in 25 dogs with AD after eating the diet for 28 days [40]. A previous unblinded, uncontrolled 8-week clinical trial in atopic dogs also found this type of enriched diet improved veterinarian Canine Atopic Dermatitis Extent and Severity Index (CADESI-03) scores and pet owner skin assessments [41]. The goal of this study is to verify those initial findings by investigating the effectiveness of a diet supplemented with vitamin C and E, polyphenols, and PUFAs in a randomized, double-blinded, placebo-controlled fashion in client owned dogs with AD.

## Results

Forty-three dogs were evaluated for the study. Three dogs were dismissed due to complete or partial response to a dietary elimination trial. Eighteen male (6 intact, 12 neutered) and 22 female dogs (5 intact, 17 spayed) with AD were enrolled over a 30-month period and randomly assigned to diet A (*n* = 20) or diet B (*n* = 20). Completion rate for the study was 100%. Diet groups did not differ by age (A = 4.3y [1.0–9.0]; B = 5.0y [2.0–11.0]), body weight (A = 14.1 kg [1.7–37.0]; B = 17.9 kg [3.9–32.6]), or gender (A = 10F, 10 M; B = 12F, 8 M). Age, breed, and gender of dogs assigned to each group are listed in Table 1. Fifteen of 20 owners feeding diet A and 17 of 20 owners feeding diet B rated the palatability as excellent. Mean change in body weight did not differ between groups with diet A losing 0.20 kg and diet B losing 0.14 kg throughout the course of the study (Table 1).

**Table 1:** Characteristics of Dogs Enrolled in Study

Dog ID	AGE (years)	SEX	START WEIGHT (kg)	END WEIGHT (kg)	BREED	GROUP
1	2	FS	5.4	5.6	Mixed	A
2	1	FS	10.9	11.3	French bulldog	A
3	8	M	7.3	7.4	Maltese	A
4	3	MN	30.0	35.0	Mixed	A
5	2	MN	8.6	8.5	Pinscher	A
6	5	FS	9.2	9.6	Schnauzer mini	A
7	8	M	37	35.1	Labrador retriever	A
8	7	F	1.7	1.6	Yorkshire terrier	A
9	9	FS	3.3	3.1	Yorkshire terrier	A
10	6	MN	14.1	13.8	French bulldog	A
11	2	FS	26.3	25.4	Spanish greyhound	A
12	4	M	36.0	34.3	Golden retriever	A
13	3	FS	28.0	28.5	Mixed	A
14	4	F	13.6	13.8	French bulldog	A
15	6	FS	12.7	14.1	French bulldog	A
16	5	M	17.8	16.8	Mixed	A
17	2	MN	33.0	34.0	German shepherd	A
18	1	FS	27.4	27.8	Dobermann	A
19	6	MN	14	14.3	Mixed	A
20	4	MN	28	28.9	American Staffordshire	A
21	2	FS	8	8.3	West highland white terrier	B
22	2	FS	28.8	27.0	Labrador retriever	B
23	11	M	17.6	16.4	Beagle	B
24	2	M	32.6	32.6	American Staffordshire	B
25	3	FS	11	11.1	French bulldog	B
26	11	M	16.9	17.5	Beagle	B
27	6	FS	19.7	19.9	American Staffordshire	B
28	3	FS	3.9	4.1	Yorkshire terrier	B
29	5	FS	29.3	29.4	Boxer	B
30	9	F	25.3	23.5	Labrador retriever	B
31	6	FS	32.4	33.2	Labrador retriever	B
32	4	MN	18.2	19.0	French bulldog	B
33	8	MN	7.2	7.4	West highland white terrier	B
34	6	FS	12.7	13.4	French bulldog	B
35	5	M	13	12.6	Andalusian bodeguero	B
36	4	FS	26	28.5	Gos D’atura Catalá	B
37	3	FS	26.8	29.2	Siberian husky	B
38	7	F	4.9	4.7	Mixed	B
39	6	MN	17	15.9	Mixed	B
40	4	MN	22	22.4	Mixed	B

Description of dogs enrolled in study. *F* intact female, *FS* female spayed, *M* intact male, *MN* male neutered

Total CADESI-4 index scores did not differ between groups on days zero, 30 or 60. Scores were not significantly different between days zero, 30 and 60 in the diet A group, but were significantly lower on day 60 in the diet B group (*P* = 0.003) (Table 2). When evaluating individual region scores of the CADESI-4 index, groups did not differ and no significant changes occurred between days. When evaluating the percentage change in CADESI-4 scores, diet B had significant reductions of 25 and 49% on days 30 and 60, respectively (*P* = 0.0007) while diet A had no change over study period. On day 60, diet B had a greater percentage change in CADESI-4 scores compared to diet A (− 48.5 (B); − 16.8 (A); *P* = 0.02) (Table 3).

**Table 2:** Total CADESI-4 index scores recorded by veterinarians during the study period

Day	Diet A	Diet B	*P* value*
0	10.5 (2.0–44.0)^a^	14.5 (4.0–29.0)^a^	0.3020
30	8.5 (1.0–24.0)^a^	10.5 (1.0–30.0)^ab^	0.2818
60	7.5 (1.0–25.0)^a^	7.0 (0.0–41.0)^b^	0.7602
*P* value^†^	0.0270	0.0034	

Total CADESI-4 index scores recorded by veterinarians during the study period. Scores did not differ between groups at any time point. Scores were significantly lower for diet B on day 60. There was no difference between days in diet A. Data are reported as median (range). *P value to compare groups within each day. †*P* value to compare days within each group. Note that for diet A, statistical significance disappeared after adjusting for multiple comparisons. Within each column, days with different superscript letters differ from each other (*p* < 0.05)

**Table 3:** Percent change in CADESI-4 index scores recorded by veterinarians during the study period

Day	Diet A	Diet B	*P* value*
30	−10.7 (−76.9–125.0)	−25.0 (−87.5–66.7)	0.4976
60	−16.8 (−87.5–275.0)	−48.5 (−100.0–57.7)	0.0212
*P* value^†^	0.1701	0.0007	

Percent change in total CADESI-4 index scores recorded by veterinarians during the study period. Diet B had significant reductions of 25 and 48.5% on days 30 and 60 while diet A had no change. On day 60, diet B had a greater percentage change in CADESI-4 scores compared to diet A. Data are reported as median (range).*P value to compare groups within each day. †*P* value to compare days within each group

Owner-reported pruritus scores did not differ between groups and both groups saw statistically significant reductions by day 15 (*P* < 0.0001). Diet B group continued to have reductions in pruritus scores with day 60 being lower than days 0,15, and 30 (Table 4). Overall, mean pruritus scores in diet B group went from 8.0 (range 4.0–10.0) to 3.5 (0.0–9.0) over the course of the study. When comparing the percent of change in owner-reported pruritus scores, diet B also performed better than diet A. By day 60, owners of dogs fed diet B reported a significant reduction (*P* < 0.0001) of 46.4% in itching, while those on diet A reported a 26.8% reduction, which was not statistically significant (*P* = 0.08). The percentage change in pruritus between groups was not statistically different on corresponding days (Table 5).

**Table 4:** Owner-reported pruritus scores

Day	Diet A	Diet B	***P*** value*
**0**	8.0 (6.0–10.0)^a^	8.0 (4.0–10.0)^a^	0.8981
**15**	7.0 (2.0–10.0)^b^	6.5 (2.0–9.0)^b^	0.4464
**30**	5.0 (1.0–9.0)^bc^	4.0 (1.0–10.0)^bc^	0.6808
**45**	5.5 (1.0–8.0)^bcd^	4.0 (1.0–8.0)^cd^	0.1872
**60**	6.0 (1.0–10.0)^bcd^	3.5 (0.0–9.0)^d^	0.1518
***P*** **value**^**†**^	< 0.0001	< 0.0001	

Owner-reported pruritus scores assessed on a scale of zero to 10 where zero is no itching and 10 is extreme itching [42]. Scores did not differ between groups and both groups saw statistically significant reductions by day 15. Diet B group continued to have reductions in pruritus scores with day 60 being lower than days 0,15, and 30. Data are reported as median (range).*P value to compare groups within each day. †*P* value to compare days within each group. Within each column, days with different superscript letters differ from each other (*p* < 0.05)

**Table 5:** Percent change in owner-reported pruritus scores

Day	Diet A	Diet B	***P*** value*
**15**	−12.5 (−77.8–33.3)	−21.1 (−50.0–16.7)^a^	0.4405
**30**	−31.0 (−87.5–50.0)	−35.4 (− 88.9–14.3)^a^	0.7616
**45**	−31.0 (− 87.5–33.3)	−42.9 (−90.0–0.0)^bc^	0.2607
**60**	−26.8 (−88.9–66.7)	−46.4 (−100.0–0.0)^c^	0.1529
***P*** **value**^**†**^	0.0815	< 0.0001	

Percent change in owner-reported pruritus scores. By day 60, diet B owners reported a significant reduction of 46.4% in itching. Data are reported as median (range).* *P* value to compare groups within each day. ^†^*P* value to compare days within each group. Within each column, days with different superscript letters differ from each other (*p* < 0.05)

## Discussion

Results of this randomized, double-blind, placebo-controlled clinical trial demonstrate significant improvement in CADESI-4 scores and owner-reported pruritus scores for dogs consuming a diet high in antioxidants, polyphenols, EPA, and DHA (diet B). This study also supports the findings of a previous uncontrolled clinical trial evaluating the same diet in dogs with AD [41]. The improvements seen in studies of diet B can be attributed to the diet’s functional ingredients. Research has demonstrated increased consumption of omega-3 fatty acids improves clinical signs associated with canine AD [6, 9]. The amount of EPA and DHA in diet B is similar to dosages used in these other clinical trials (1.35 g/1000 kcal) when dogs meet daily energy requirements as estimated by the National Research Council (95–180 × kg0.75) [43]. The antioxidant vitamin E has also demonstrated effectiveness in lowering AD CADESI scores when supplemented at levels similar to diet B 37. While studies evaluating the role of polyphenols in canine AD are lacking, research utilizing rodent models of AD suggest resveratrol and quercetin can mitigate clinical signs and improve skin health [44, 45].

The control diet chosen in this study provides a similar macronutrient profile to the test diet while being lower in key nutrients (n-3 fatty acids and vitamin E) and functional ingredients high in polyphenols and antioxidants. While the total n-3 fatty acid concentration of the test diet was nearly 6 times higher than the control, the composition of n-3 fatty acids in the control diet was not measured. Although comparing individual nutrient changes in a clinical trial is ideal, for cost and efficiency the authors felt it was reasonable to initially evaluate the composite effects of test diet to determine efficacy in AD. Additional studies assessing individual ingredient and nutrient effects would be beneficial.

Owner-reported pruritus and CADESI-4 scores trended lower over time in both diet groups. Some of this improvement may be attributed to a placebo effect and/or treatment with oclacitinib and parasite preventatives. However, diet B owners noted 46% reduction in pruritus while the 27% reduction noted by diet A owners was not statistically different from baseline. The CADESI-4 scores were not statistically lower than baseline for diet A, but diet B showed improved scores on day 60 with a drop of almost 50%. The dramatic improvement in both veterinarian and owner-reported AD scores implies a diet effect, rather than improvement from medical therapy alone. In conclusion, this clinical trial demonstrates feeding a diet enriched with ingredients to improve skin health and reduce inflammation improves the clinical signs of AD in dogs.

## Conclusion

Compared to control, a diet enriched with ingredients to improve skin health and reduce inflammation improve the clinical signs of AD in dogs as measured by pet owners and veterinarians.

## Methods

This prospective study was approved by the ethics committee of Complutense University. Dogs with AD examined at our veterinary hospital were included in this study. Dogs were diagnosed with AD based on history, clinical signs, and fulfillment of Favrot’s criteria [46]. Patients also had to be free from ectopic parasites as determined by deep and superficial skin scraping and have received parasite preventative medications for at least 2 months prior to enrollment. Primary pyodermas were excluded through tape cytology and culture of pustules if present. Once ectopic parasites and pyodermas were excluded, patients underwent an 8-week dietary elimination trial with a hydrolyzed protein diet to rule-out food allergic dermatitis (Hill’s Prescription Diet™ z/d™, Hill’s Pet Nutrition, Topeka, KS, USA).

Oclacitinib (Apoquel®, Zoetis, Parsippany, NJ, USA) treatment was not permitted before enrollment, but once admitted to the study oclacitinib was initiated at a dose of 0.4–0.6 mg/kg/ every 12 h for 14 days; followed by a maintenance dose of 0.4–0.6 mg/kg every 12 h until the end of the study. This treatment was initiated in all dogs since dietary intervention was viewed as an adjunct, rather than replacement, to medical therapy, and oclacitinib is considered the standard of care. Patients were also encouraged to wear a fly and flea preventative collar throughout the study but were prohibited from giving oral parasite preventatives. Owners were not permitted to give their dogs dietary supplements, treats, or topical medications during the treatment period.

Dogs meeting enrollment criteria were randomly assigned to a control diet A (Purina Proplan™ Opti Nutrition Adult™ Medium dry, Nestlé Purina, St. Louis, MO, USA) or test diet B (Hill’s Prescription Diet™ Derm Defense™ dry, Hill’s Pet Nutrition, Topeka, KS, USA) for 60-days (Table 6). Diet A ingredients included dehydrated poultry protein, wheat, maize, chicken, animal fat, dried beet pulp, soya meal, maize grits, rice, digest, gluten, minerals, and fish oil. Diet B ingredients included maize, brewer’s rice, chicken and turkey meal, dried whole egg, soybean oil, flaxseed oil, animal fat, dried beet pulp, fish oil, minerals, vitamins, taurine, beta-carotene, and mixed tocopherols. Diet B also contained an antioxidant blend with rosemary, green tea, citrus pulp, and vitamin C. (Table 7). The control diet was chosen because it is a commonly used and widely available dog food in Spain. The control diet also had a similar macronutrient profile to the test diet, while containing lower concentrations of omega-3 fatty acids, polyphenols, and the antioxidants vitamin C and E. Both dog owners and investigators were blinded to the diets. Dog owners were provided feeding amounts in grams for each diet based on their pet’s current body weight. They were also instructed to adjust the food amount as necessary to maintain optimal weight.

**Table 6:** Nutrient composition of study diets

Nutrient	Diet A DMB	Diet A per 1000 kcal	Diet B DMB	Diet B Per 1000 kcal
Crude protein	25.0%	62 g	22.7%	55 g
Crude fat	15.0%	37 g	17.0%	41 g
Nitrogen-free extract	40.5%	100 g	53.4%	130 g
Crude fiber	2.5%	6.2 g	1.6%	4 g
Linoleic acid	n/a	n/a	4.59%	11.2 g
alpha-Linolenic acid	n/a	n/a	1.11%	2.7 g
Eicosapentaenoic + Docosahexaenoic acid	n/a	n/a	0.56%	1.38 g
Eicosapentaenoic acid	n/a	n/a	0.33%	0.81 g
Docosahexaenoic acid	n/a	n/a	0.23%	0.57 g
Total n-6 fatty acids	2.6%	6.4 g	4.76%	11.6 g
Total n-3 fatty acids	0.3%	0.74 g	1.81%	4.42 g
Zinc	187 mg/kg	0.045 g	267 mg/kg	0.065 g
Vitamin A	20,710 IU/kg	5123 IU	10,638 IU/kg	2590 IU
Vitamin E	473 mg/kg	0.12 g	874 mg/kg	0.21 g
Vitamin C	70 mg/kg	0.017 g	98 mg/kg	0.024 g
Beta carotene	n/a		1.6 mg/kg	0.4 mg
Energy (kcal)	4042/kg	3840/kg as fed	4110/kg	3760/kg as fed
Energy (kJ)	16,911/kg	16,066/kg	17,200/kg	15,730/kg

Legend: Nutrient composition of test diets on a dry matter basis (DMB) and per 1000 kcal basis

Metabolizable energy and resultant nutrient concentration per 1000 kcal were calculated based on modified Atwater factors (protein 3.5 kcal/g; carbohydrate 3.5 kcal/g; fat 8.5 kcal/g)

**Table 7:** Classification of major functional dietary ingredients in diet B

Polyunsaturated Fatty Acids	Polyphenol	Antioxidant
Soybean oil	Flaxseed	Green tea
Flaxseed	Brewer’s rice	Brewer’s rice
Fish oil	Rosemary	Dried beet pulp
	Green tea	Rosemary
	Citrus pulp	Vitamin E
		Vitamin C
		Beta-carotene

Legend: Classification of functional dietary ingredients in test diet B [47–50]

The severity of AD was assessed by a single investigator who was blinded to diet assignment. Assessments occurred at days zero, 30, and 60 using the CADESI-4 index which rates the severity and extent of the skin lesions (erythema, lichenification and alopecia/excoriation) on a scale from 0 to 3 at 20 anatomic locations [42]. CADESI-4 scores of less than 8 are considered normal [42]. Owners also assessed intensity of their pet’s pruritus on days zero,15, 30, 45, and 60 using a previously validated scale of zero to 10 where zero is no itching and 10 is extreme itching [51]. Itching scores of less than 3.5 are considered normal [51].

### Statistical methods

Normal probability plots showed that dermatological scores performed by veterinarians (total score over the entire dog and for each of the 20 regions within each dog), percent change from baseline for the dermatological scores, age, and body weight were skewed. Accordingly, numerical data were summarized as medians with a range while categorical data (sex, breed, and final assessment [positive or negative]) were summarized as counts and percentages. Baseline prognostic factors were compared between diet groups using the Wilcoxon rank sum test (age and body weight) and Fisher’s exact test (sex and breed).

Effects of diet (A vs B) and day (0 vs 30 vs 60) on the primary outcomes (total dermatological score and percent change in total dermatological core) were assessed using linear general estimating equations (GEE). Each of the linear models specified diet, day, and the interaction between diet and day as fixed effects. Correlations between observations within dog (the blocking factor) were modeled by specifying a compound symmetry covariance matrix. The interaction between diet and day was further analyzed (sliced) to extract comparisons between diets within each day and comparisons between days within each diet. *P* values for 2-way comparisons between days within diet were adjusted for multiple comparisons using Tukey’s procedure.

For region specific dermatological scores (and their percent change from baseline), diet groups were compared within each day using the Wilcoxon rank sum test, while the days were compared within diet using Friedman’s chi-square test. *P* values for 2-way comparisons between days within diet were adjusted for multiple comparisons using Bonferroni’s procedure. When computing percent change from baseline, a small value of 0.01 was added to each of the region-specific scores to avoid undefined values for dogs with a baseline value of 0. An overall outcome of positive or negative was assigned to each dog when the study concluded. This outcome was compared between diet groups using Fisher’s exact test. Statistical significance was set at α = 0.05. All analyses were performed using SAS version 9.4 (Cary, NC, USA).

## Data Availability

All data generated or analysed during this study are included in this published article [and its supplementary information files].

## Abbreviations

AD: Atopic dermatitis
Ig: Immunoglobulin
PUFA: Polyunsaturated fatty acid
DHA: Docosahexaenoic acid
EPA: Eicosapentaenoic acid
CADESI: Canine Atopic Dermatitis Extent and Severity Index
